# Genetic Variations in Bitter Taste Receptors and COVID-19 in the Canadian Longitudinal Study on Aging †

**DOI:** 10.3390/biomedicines13102473

**Published:** 2025-10-11

**Authors:** Marziyeh Shafizadeh, Mohd Wasif Khan, Britt Drögemöller, Chrysi Stavropoulou, Philip St. John, Rajinder P. Bhullar, Prashen Chelikani, Carol A. Hitchon

**Affiliations:** 1 Department of Oral Biology and Manitoba Chemosensory Biology Research Group, Dr. Gerald Niznick College of Dentistry, Rady Faculty of Health Sciences, University of Manitoba, Winnipeg, MB R3E 0W2, Canada; shafizam@myumanitoba.ca (M.S.); rajinder.bhullar@umanitoba.ca (R.P.B.); prashen.chelikani@umanitoba.ca (P.C.); 2 Children’s Hospital Research Institute of Manitoba, University of Manitoba, Winnipeg, MB R3E 3P4, Canada; khanmw@myumanitoba.ca (M.W.K.); britt.drogemoller@umanitoba.ca (B.D.); 3 Department of Biochemistry and Medical Genetics, Max Rady College of Medicine, Rady Faculty of Health Sciences, University of Manitoba, Winnipeg, MB R3E 0J9, Canada; 4 Paul Albrechtsen Research Institute CancerCare Manitoba, Winnipeg, MB R3E 0V9, Canada; 5 Centre on Aging, University of Manitoba, Winnipeg, MB R3T 2N2, Canada; 6 Department of Dental Diagnostic and Surgical Sciences, Dr. Gerald Niznick College of Dentistry, Rady Faculty of Health Sciences, University of Manitoba, Winnipeg, MB R3E 0W2, Canada; chrysi.stavropoulou@umanitoba.ca; 7 Department of Internal Medicine, Max Rady College of Medicine, Rady Faculty of Health Sciences, University of Manitoba, Winnipeg, MB R3A 1R9, Canada; pstjohn@hsc.mb.ca

**Keywords:** CLSA, COVID-19, taste receptors, TR2, TAS2R, single nucleotide polymorphisms, genetics

## Abstract

**Background/Objectives**: Bitter Taste Receptors (encoded by *TAS2R* genes) are expressed in mucosal and bronchial epithelia, as well as in immune cells, contributing to defense against airborne pathogens such as SARS-CoV-2. Data on single-nucleotide polymorphisms (SNPs) in *TAS2R* genes or pseudogenes in COVID-19 are limited. This study examined the association between *TAS2R* SNPs and COVID-19 infection and seroconversion in European individuals participating in the Canadian Longitudinal Study on Aging. **Methods**: Data from the Genome-wide Genetic Data, Comprehensive Baseline (version 7.0), Follow-up 2 (version 1.1), COVID-19 Questionnaire Study (4-2020 to 12-2020), and COVID-19 Seroprevalence (Antibody) Study (11-2020 to 7-2021) datasets were accessed. Associations of *TAS2R* SNPS with COVID-19 infection or seroconversion were determined using logistic regression adjusted for sociodemographics, genetic principal components, smoking, vaccine doses, and chronic medical conditions (diabetes, immune-mediated inflammatory diseases (IMIDs), respiratory disease, and cardiovascular disease). **Results**: In the COVID-19 Questionnaire Study (N = 14,073), the rs117458236 (C) variant in *TAS2R20* showed a trend toward an association with COVID-19 infection (OR = 1.95; 95% Confidence Interval (CI): 0.98, 3.51). In the COVID-19 Antibody Study (N = 8313), the rs2234235(G) variant in *TAS2R1* was associated with anti-nucleocapsid (OR = 1.55; CI: 1.06, 2.20) and anti-spike response (OR = 0.74; CI: 0.57, 0.98); the rs2234010(A) variant in *TAS2R5* was associated with anti-nucleocapsid (OR = 1.56; CI: 1.08, 2.19); and the rs34039200(A) variant in *TAS2R62P* was associated with anti-spike (OR = 0.86; CI: 0.77, 0.97). In a subgroup analysis, the rs2234235(G) variant in *TAS2R1* was associated with a decreased anti-spike response to infection or vaccination in individuals with IMIDs or respiratory disease and an increased risk of SARS-CoV-2 infection. **Conclusions**: *TAS2R* variants are associated with COVID-19 infection and vaccine response. These data may inform personalized management and vaccination strategies.

## 1. Introduction

Coronavirus disease 2019 (COVID-19) has affected over 777 million individuals and caused more than 7 million deaths worldwide [1]. Despite the availability of effective vaccines and the subsequent lifting of public health restrictions, COVID-19 remains a significant public health concern. Globally, over 65,000 new cases and 3500 deaths are reported each month, posing particular risks to older adults and individuals with compromised immune systems [1,2]. This underscores the importance of understanding the factors that influence susceptibility to, and the immune response against, COVID-19.

The innate immune response in the nasopharyngeal and oral mucosa plays a crucial role in the early defense against airborne pathogens, including severe acute respiratory syndrome coronavirus 2 (SARS-CoV-2): the virus that causes COVID-19. The receptors required for SARS-CoV-2 binding and cell entry are expressed in the nasal and salivary epithelia [3]. Additionally, nasopharyngeal tissues and salivary glands may serve as potential reservoirs for the virus [4,5,6,7].

Bitter Taste Receptors (T2Rs) are G-protein-coupled receptors encoded by *TAS2R* genes, as designated by the HUGO Gene Nomenclature Committee [8]. T2Rs are expressed in taste buds and in various tissues throughout the body, including mucosal epithelia and immune cells [9,10,11]. They contribute to airway reflex protection, increased ciliary movement, and bronchodilation [12,13,14,15,16,17]. These effects are mediated, in part, by stimulating mucosal cells to release nitric oxide and calcium-mediated antimicrobial peptides [18,19,20]. Nitric oxide inhibits SARS-CoV-2 replication by impairing the interaction between the viral spike protein and host receptors and reducing viral RNA production [21]. Taste loss, a common symptom in COVID-19 patients, with 39.2% reporting taste dysfunction [22], may be partially mediated through T2Rs. Antimicrobial peptides inhibit viral activity through mechanisms such as destabilizing the viral envelope [23]. Finally, T2Rs in lung macrophages inhibit the release of pro-inflammatory cytokines, potentially limiting the damage caused by an excessive immune response to the virus [24].

Given these roles, variations in *TAS2R* genes may impact susceptibility to COVID-19 and the immune response to infection or vaccination. Identifying *TAS2R* genetic variants associated with COVID-19 outcomes could provide insights into more effective preventive protocols and help identify individuals who may require early interventions, including those with chronic medical conditions that increase their risk [2,25,26,27,28]. Moreover, understanding the relationship between *TAS2R* variants and serological response to SARS-CoV-2 could contribute to the development of personalized vaccination strategies.

Humans have 25 *TAS2R* genes and 12 non-functional *TAS2R* pseudogenes. Pseudogenes resemble functional genes but cannot encode functional proteins due to structural disruptions; however, they can be transcribed into RNA and impact gene expression regulation [29,30]. A variety of single-nucleotide polymorphisms (SNPs; single-nucleotide variations in the DNA sequence) in *TAS2R* genes can affect receptor function [31]. Among the 37 human *TAS2R* genes, *TAS2R38* is the most widely studied. It contains three SNPs in linkage disequilibrium (genetic variants that are inherited together; rs1726866, rs713598, and rs10246939), resulting in two common haplotypes. The functional haplotype encodes proline-49, alanine-262, and valine-296 (PAV), while the non-functional haplotype encodes alanine-49, valine-262, and isoleucine-296 (AVI). The “supertaster” phenotype (PAV/PAV) is associated with the higher expression of nitric oxide and antimicrobial peptides, as well as a lower risk of chronic rhinosinusitis compared to the non-taster phenotype (AVI/AVI) [32,33]. Few studies have assessed the association between *TAS2R* genetic variants and the risk or severity of COVID-19 [34,35,36,37]. Studies examining these associations have largely focused on *TAS2R38* but have not accounted for high-risk groups, such as those with chronic medical conditions, and have reported inconsistent findings. Moreover, *TAS2R38* has relatively low expression in extra-oral tissues, suggesting that other *TAS2R* genes with higher expression in extra-oral tissues may have a stronger association with COVID-19 outcomes [9]. To date, no research has examined the association between COVID-19 outcomes and other *TAS2R* genes or pseudogenes. Additionally, no published data exist on the association between *TAS2R* variants and SARS-CoV-2 seroconversion, defined as the development of detectable antibodies in the blood following infection or vaccination.

This study aimed to evaluate the association between *TAS2R* SNPs and COVID-19 outcomes, including seroconversion, and to determine whether this association differs in high-risk groups within the Canadian Longitudinal Study on Aging (CLSA). Our primary objective was to evaluate the association between *TAS2R* SNPs and COVID-19 infection. The second objective was to assess the relationship between *TAS2R* SNPs and post-infection and post-vaccination antibody response. Finally, we aimed to determine whether these associations with COVID-19 outcomes and seroconversion differ among individuals with or without chronic medical conditions known to increase the risk of severe COVID-19 infection or reduce seroconversion, including diabetes, cardiovascular disease, respiratory disease, and immune-mediated inflammatory disorders (IMIDs).

## 2. Materials and Methods

### 2.1. Study Population and Data Preprocessing

The CLSA is a longitudinal national cohort of 50,000 individuals designed to evaluate the influence of various factors on health and disease [38]. We obtained access to the CLSA Genome-wide Genetic Data Release (version 3), Comprehensive Baseline Dataset (version 7.0), Comprehensive Follow-up 2 Dataset (version 1.1), COVID-19 Questionnaire Study (data collected from April 2020 to December 2020), and COVID-19 Seroprevalence (Antibody) Study (data collected from November 2020 to July 2021), under CLSA Application ID: 23CA016. This study was approved by the University of Manitoba’s Health Research Ethics Board (HREB HS26021-H2023:173).

The CLSA methods, including eligibility criteria, sampling, data collection, and processing, are explained in detail in the Researchers section of the website (https://www.clsa-elcv.ca/). In summary, individuals aged 45 to 85 were enrolled from various locations in Canada, and using standard protocols, information was collected, including demographic, lifestyle, and behavioral data. In the COVID-19 Questionnaire and Seroprevalence (Antibody) studies, additional data was collected on COVID-19 infection, vaccination, and serology (nucleocapsid and spike antibodies). COVID-19 Questionnaire data was collected weekly/biweekly and monthly using web-based and telephone-based interviews and included information on prior COVID-19 infection. The COVID-19 Seroprevalence dataset included data on the number, date and results of prior COVID-19 tests. All serology tests were performed over 14 days following a reported COVID-19 test, with the shortest interval being 20 days. First vaccination ranged from 115 days prior to serology collection to 117 days post serology collection. Only 310 (4%) of participants had a serology sample collected within 2 weeks of first vaccination.

A subset of 26,622 participants provided consent to collect their blood samples for genotyping. The genetic dataset comprises genotypes for 794,409 variants, obtained using the Affymetrix Axiom array, in addition to 308 million imputed variants from the TOPMed reference panel [39]. SNPs within *TAS2R* genes were extracted using PLINK (v1.9) [40] based on their GRCh38 coordinates retrieved from the National Center for Biotechnology Information (NCBI) Gene database [41]. Annotations were retrieved using the Ensembl Variant Effect Predictor (VEP) [42].

### 2.2. Eligibility Criteria

We used PLINK (v.1.9) for sample and marker quality control based on previously established protocols [39,43]. The exclusion criteria for genetic markers were as follows: inconsistencies in genotype frequency between control replicates and batches; deviation from the Hardy–Weinberg equilibrium (*p* < 3.15 × 10^−10^); genotype missingness exceeding 5%; minor allele frequency (MAF; the prevalence of the less common allele of a SNP within the study population) ≤ 0.01; and insertions/deletions (indels). Samples were excluded based on the following: heterozygosity outliers; familial relatedness of at least third-degree (assessed by identity-by-descent analysis); mismatches between chromosomal and self-reported sex; and more than 5% missing genotypes. Additionally, imputed variants with a quality score >0.8 were included [44]. To account for population stratification, only individuals with European ancestry were included, who were previously identified by the CLSA based on genetic principal component analysis (PCA) [39].

### 2.3. COVID-19 Infection

Data from the CLSA COVID-19 Questionnaire Study were used to assess the association between *TAS2R* variants and COVID-19 infection. Data were collected at baseline, weekly, biweekly, monthly, and at the time of exit. Two outcomes were evaluated: A. Confirmed infection: Participants who reported a positive nucleic acid amplification test for COVID-19. B. Probable/confirmed infection: Participants who either reported a positive nucleic acid amplification test or were told by a healthcare professional that they had COVID-19 without a confirmed test. The probable cases were included, as many individuals were not tested in the early pandemic [45]. Individuals were coded as positive for infection if they reported at least one positive response at any time point during the questionnaire; otherwise, they were coded as negative. Those with missing data at all time points were excluded from the analysis.

### 2.4. COVID-19 Antibodies

The CLSA COVID-19 Seroprevalence (Antibody) Study was used to evaluate the association between *TAS2R* variants and the presence of nucleocapsid antibody (a marker of serologically confirmed COVID-19 infection) and spike antibody (anti-receptor binding domain, a marker of COVID-19 infection or vaccine seroconversion). To assess the relationship between *TAS2R* SNPs and the serological response to COVID-19 vaccination, we focused on participants who had received at least one vaccine dose and showed no evidence of prior infection (i.e., no detectable nucleocapsid antibody). Within this subgroup, we investigated the association between *TAS2R* variants and the presence of spike antibody, defining cases as individuals with a positive spike antibody status and controls as those with a negative spike antibody status.

### 2.5. Case Definition for Chronic Medical Conditions

Diabetes, respiratory disease, and cardiovascular conditions were included in logistic regression models as covariates and in subgroup analyses since they are associated with increased risk of severe COVID-19 infection and are among the most common chronic conditions in Canada [46,47]. Immunocompromised individuals, such as those with immune-mediated inflammatory diseases (IMIDs), are also at a higher risk of severe COVID-19 infection or poorer vaccine-induced immunogenicity, potentially due to immune dysregulation or the use of immunomodulatory medications [27,28,48,49]. Among IMIDs, we focused on rheumatoid arthritis and inflammatory bowel disease, due to their high prevalence in Canada, overlapping use of immunomodulatory agents, and evidence of association with COVID-19 severity [50,51,52,53,54,55]. We generated case definitions for these conditions using self-reported data and medication use, based on algorithms developed through an extensive literature review (Figure S1 and Table S1) [56,57,58,59,60,61,62,63,64,65,66,67,68,69]. To confirm the relative accuracy of the definitions, we compared the prevalences for each condition with those reported by the Canadian Chronic Disease Surveillance System (CCDSS) and other CLSA publications [59,65,68,70,71].

### 2.6. Statistical Analysis

Study population characteristics were summarized using the gtsummary package in R. *TAS2R* variants were initially tested in univariable models with chi-squared tests using PLINK (v.1.9) to identify variants associated with the outcomes of interest (COVID-19 infection; COVID-19 seroconversion). Independent SNPs (variants with very low correlation, r^2^ < 0.1 within a 1000 kb genomic window, N = 35) [39] were then selected for inclusion in multivariable logistic regression under an additive genetic model. The list of covariates that were initially included is provided in Table S2. Assessment of multicollinearity using Pearson correlation analysis revealed no covariate pairs exceeding 0.6; therefore, all covariates were retained for further analysis (Figure S2). Stepwise variable selection was then applied to identify the best-fitting models, and the selected variables were included in the final analysis. The final models included the following variables: age, sex, top 10 principal components for the European ancestry subset released by the CLSA (ePC1-10), smoking status, dwelling area (available only in the Questionnaire Study), dwelling ownership, number of household residents, number of vaccine doses (available only in the Antibody Study), post-secondary education, diabetes, respiratory disease, cardiovascular disease, and IMID. In the COVID-19 Questionnaire analyses, dwelling ownership and number of household residents were excluded due to a high rate of missing data. Individuals with missing data were excluded from the logistic regression analyses.

The analyses were conducted separately using data from the COVID-19 Questionnaire and Seroprevalence (Antibody) studies, as these datasets include overlapping but nonidentical populations. Subgroup analyses were performed for each outcome, stratifying individuals based on the presence or absence of chronic medical conditions. Statistical significance was set at *p* <0.05, and Bonferroni correction was applied to adjust for multiple comparisons, considering the number of independent outcomes as 1 due to the high correlation between outcomes (Phi coefficient > 0.1) [72]. Odds Ratios (ORs) with 95% Confidence Interval (CI) are reported. To explore the potential functional mechanisms of the associated variants, we queried RegulomeDB v2.2 and HaploReg v4.2, and used the RNAsnp web server to predict their effects on mRNA secondary structures [73,74,75].

## 3. Results

After applying the eligibility criteria, 14,073 individuals from the COVID-19 Questionnaire Study and 8313 from the COVID-19 Antibody Study were included. Population characteristics are summarized in Table 1.

**Figure 1:**
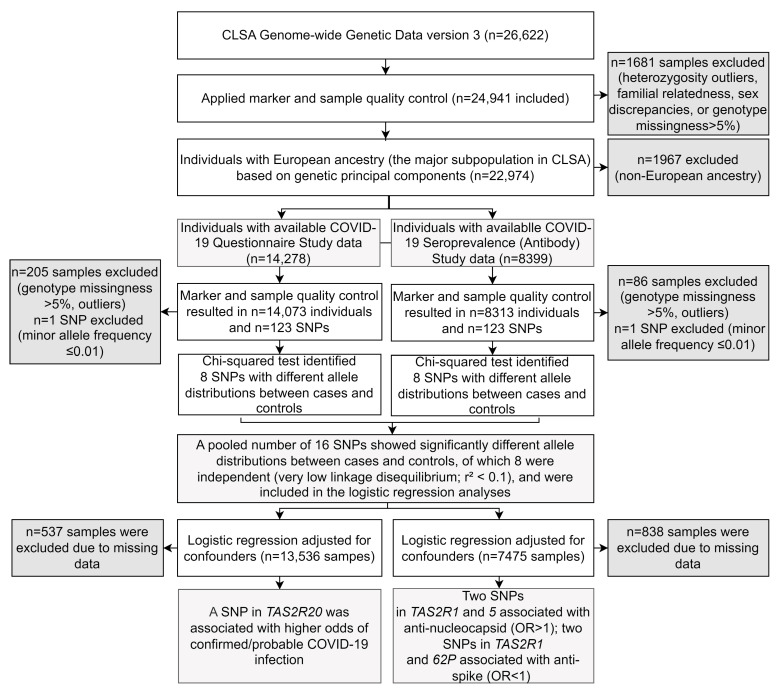
Overview of sample selection, data analysis, and results.

Figure 1 provides an overview of the sample selection, data analysis, and results.

Chi-squared tests identified 16 SNPs with differing allele distributions between cases and controls (Table S3). Among these, eight SNPs were independent (r^2^ < 0.1) and were included in logistic regression analyses.

### 3.1. CLSA COVID-19 Questionnaire Study

In the Questionnaire Study, 2299 (16.33%) participants were tested for COVID-19, with 45 (1.96% of those tested) having a positive result (confirmed cases). The total number of confirmed/probable cases was 153 (1.09% of the cohort). Confirmed/probable COVID-19 infection was significantly associated with age (OR: 0.97; 95% Confidence Interval (CI): 0.95, 0.99; *p* < 0.001), female sex (OR: 1.80; CI: 1.28, 2.58; *p* < 0.001), rental dwelling status (OR: 1.66; CI: 1.08, 2.47; *p* = 0.017), and respiratory disease (OR: 1.78; CI: 1.24, 2.52; *p* = 0.001) (Table 2). The rs117458236 variant, located in the 3′ untranslated region (UTR) of *TAS2R20*, was associated with a trend toward increased odds of confirmed/probable infection (OR: 1.95, CI: 0.98, 3.51; *p* = 0.039). The RNAsnp predicted minimal changes in mRNA secondary structure and base-pairing probabilities (Figure S3). HaploReg v4.2 indicated that this variant alters binding sites for immune-related transcription factors (Ik-2, NF-AT, and NF-AT1), with higher predicted affinity for the T allele, suggesting a potential regulatory effect. No significant association was found between confirmed infection and other *TAS2R* variants (Table 2).

### 3.2. CLSA COVID-19 Seroprevalence (Antibody) Study

Vaccination history and antibody response data are summarized in Table 3. A total of 370 (4.45%) participants had detectable nucleocapsid antibody, indicating serologically confirmed infection, while 3773 had detectable spike antibody, which could result from infection or vaccination. Of the 310 samples collected within 2 weeks of first vaccination, 222 (76%) or 3% total cohort were negative for spike antibody.

Logistic regression results are summarized in Table 4. Spike antibody was positively associated with female sex (OR: 1.25; CI: 1.08, 1.43; *p* = 0.002), number of vaccine doses (OR: 90.2; CI: 72.6, 114; *p* < 0.001), and negatively associated with IMID diagnosis (OR: 0.65; CI: 0.49, 9.85; *p* = 0.003). Three *TAS2R* variants were associated with antibody response: rs2234235, a synonymous variant in *TAS2R1*, with higher odds of nucleocapsid antibody (OR: 1.55; CI: 1.06, 2.20; *p* = 0.018) and lower odds of spike antibody (OR: 0.74; CI: 0.57, 0.98; *p* = 0.033); rs2234010, located in the 5′ UTR in *TAS2R5*, with higher odds of nucleocapsid antibody (OR: 1.56; CI: 1.08, 2.19; *p* = 0.014); and rs34039200 in *TAS2R62P* (pseudogene) with lower odds of spike antibody (OR: 0.86; CI: 0.77, 0.97; *p* = 0.013). The associations of rs2234010 with higher odds of nucleocapsid antibody and rs34039200 with lower odds of spike antibody remained significant after applying a more conservative *p* value estimate (*p* < 0.017) adjusted for the 3 statistically important comparisons.

RNA secondary structure prediction using RNAsnp revealed subtle changes in mRNA stability between alleles of rs2234235 in *TAS2R1* (Figure S3) [75]. RegulomeDB indicated that this variant maps to quiescent or heterochromatic regions in immune cells, including thymus and T/B lymphocytes, suggesting low basal regulatory activity [73]. RNAsnp predicted significant changes in mRNA secondary structure for the rs2234010 variant in *TAS2R5* (Figure S3). Ensembl VEP annotated rs34039200 as a non-coding exon variant in the *TAS2R62P* pseudogene, overlapping several lncRNAs such as EPHA1-AS1 [42]. RegulomeDB placed this variant in enhancer-like regions across immune cells and embryonic stem cells, indicating potential regulatory activity.

Among the 8313 participants, 4267 had received at least one vaccine dose, with Pfizer-BioNTech being the most commonly administered (N = 3901). Logistic regression in vaccinated individuals without detectable nucleocapsid antibody (Table 5) confirmed the association of rs2234235 in *TAS2R1* and rs34039200 in *TAS2R62P* with lower odds of spike antibody in vaccinated individuals (OR: 0.71; CI: 0.54, 0.95; *p* = 0.021; OR: 0.86; CI: 0.76, 0.97; *p* = 0.017, respectively).

### 3.3. Subgroup Analysis

In the Questionnaire Study, subgroup analysis was performed for confirmed/probable infection due to the larger sample size (Table S4). The rs117458236 variant in *TAS2R20* remained associated with infection in individuals with diabetes (OR: 3.27; CI: 0.93, 8.91; *p* = 0.035) or cardiovascular disease (OR: 2.69; CI: 1.10, 5.65; *p* = 0.016). Furthermore, this variant was associated with an increased risk of infection in individuals without IMID (OR: 2.15; CI: 1.08, 3.88; *p* = 0.018), but not in those with IMID. The rs2234235 in *TAS2R1* was linked to confirmed/probable infection in individuals with respiratory disease (OR: 2.65; CI: 1.08, 5.61; *p* = 0.018) or IMID (OR: 7.39; 95% CI: 1.36, 33.8; *p* = 0.012), as well as in those without diabetes (OR: 2.08; CI: 1.11, 3.57; *p* = 0.013). rs1726866, a missense variant (Val 262 Ala) in *TAS2R38,* was associated with infection in individuals with respiratory disease (OR: 1.86; CI: 1.22, 2.88; *p* = 0.004).

In the Antibody Study, a subgroup analysis confirmed the association of rs2234235 in TAS2R1 and rs2234010 in TAS2R5 with nucleocapsid antibody (OR > 1; *p* < 0.05) in most healthy subgroups (Table S5). Additionally, rs77837442 in TAS2R19 was associated with reduced anti-nucleocapsid antibody levels in individuals without diabetes (OR: 0.23; CI: 0.04, 0.71; *p* = 0.036). No significant association was found with other variants. The association of rs34039200 in TAS2R62P with spike antibody remained significant in individuals with diabetes (OR: 0.72; CI: 0.56, 0.92; *p* < 0.01), as well as in those without respiratory disease or IMID (Table S6). Although rs2234235 in TAS2R1 was associated with the spike antibody in the entire cohort (OR: 0.74; CI: 0.57, 0.98; *p* = 0.033), a subgroup analysis revealed a significant association only in patients with respiratory disease (OR: 0.48; CI: 0.27, 0.85; *p* = 0.012) and with a trend toward significance in patients with IMID (OR: 0.37; CI: 0.13, 1.04; *p* = 0.058). The two SNPs in the 5′ UTR of TAS2R5, which were not associated with spike antibody in the overall cohort, showed a significant association in patients with diabetes (OR: 2.19; CI: 1.17, 4.24; OR: 2.15; CI: 1.13, 4.16).

## 4. Discussion

This study examined the relationship between TAS2R genetic variants and COVID-19 outcomes in individuals of European ancestry participating in the CLSA cohort. The prevalence of confirmed/probable COVID-19 infection (1.09%) and serologically confirmed infection (4.45%) was consistent with findings from other studies on the CLSA dataset [37,60,76,77]. One allele in *TAS2R20* was associated with a trend to an increased risk of confirmed or probable infection. Additionally, two alleles in *TAS2R1* and *TAS2R5* were associated with the presence of nucleocapsid antibody, and two alleles in *TAS2R1* and *TAS2R62P* were linked to lower odds of spike antibody seroconversion following COVID-19 vaccination or infection.

This study builds upon prior studies assessing TAS2R variants and COVID-19 outcomes, with a specific focus on *TAS2R38* [34,35,36,37]. These prior studies primarily focused on three variants (rs1726866, rs713598, and rs10246939) that give rise to the two common haplotypes. Research has focused on these variants because the three TAS2R38 phenotypes—supertaster (PAV/PAV), taster (PAV/AVI), and non-taster (AVI/AVI)—can be clinically assessed using taste strip testing. A recent study using CLSA data found no significant association between *TAS2R38* haplotypes and COVID-19 infection or symptoms, aligning with the findings of this study on *TAS2R38* [37,72]. Similarly, Risso et al. (2022) found no significant association between the *TAS2R38* phenotype and either the presence or severity of SARS-CoV-2 infection [36]. In contrast, Barham et al. reported an association between the nontaster phenotype and more severe COVID-19 symptoms in a cohort of 100 patients [78]. Similarly, Parsa et al. in a 2021 systematic review and meta-analysis, found the PAV haplotype (C allele in rs10246939) to be associated with lower COVID-19 mortality [35]. Another study by Barham et al. on a cohort of 1935 individuals found that non-tasters were more likely to test positive for COVID-19, need hospitalization after infection, and experience prolonged symptom duration [34].

The SNP associated with COVID-19 infection identified in this study was located in *TAS2R20*. Additionally, rs3851584 in *TAS2R14* showed a borderline significant association with the infection (*p* = 0.058). *TAS2Rs* are expressed in the bronchial epithelium and airway smooth muscles, where they play a role in airway clearance [12,13,14,15,16,17]. Additionally, they are expressed in immune cells, such as macrophages, monocytes, and neutrophils, and contribute to the innate immune response against viral infections through nitric oxide production, calcium-mediated antimicrobial peptides, and enhanced phagocytosis [18,19,20,21,79]. *TAS2R20* and *TAS2R14* exhibit the highest expression levels in the bronchial epithelium [9], which may explain the association with SARS-CoV-2 infection. The rs117458236 variant associated with infection is located in the 3′ untranslated region (UTR) of *TAS2R20*. Genetic variants in UTRs can influence post-transcriptional gene regulation by affecting mRNA secondary structure, stability, translation efficiency, and localization [80,81]. Although none of the variants associated with seroconversion alter T2R protein sequences, functional annotations from RegulomeDB and RNAsnp suggest regulatory potential. Notably, rs2234010 in *TAS2R5* is predicted to modify mRNA stability and secondary structure, potentially affecting post-transcriptional gene expression (Figure S3). In addition, rs34039200, despite being located in the *TAS2R62P* pseudogene, overlaps enhancer chromatin and may exert regulatory effects via lncRNA-mediated mechanisms. Collectively, the associated variants may influence COVID-19 infection and antibody seroconversion through non-coding regulatory mechanisms.

*TAS2R* polymorphisms have been associated with various clinical conditions, although the underlying biological mechanisms are yet to be elucidated [82]. *TAS2R38* genotypes have been associated with the abundance of buccal bacterial species linked to rheumatoid arthritis [83]. Variants in *TAS2R3*, *TAS2R4*, *TAS2R5*, and *TAS2R60* have also been associated with dental plaque microbial abundance in severe early childhood caries [84]. Machine learning analyses suggest that *Streptococcus mutans*, a cariogenic bacterium, may act as a mediator between *TAS2R* variants and early childhood caries [85]. Quorum-sensing molecules released by *S. mutans* are detected by the T2R family [86], and infection of gingival epithelial cells with *S. mutans* induces T2R14-dependent IL-8 secretion [87]. T2Rs further modulate innate immune responses, including autophagy, in response to S. mutans [88,89]. Beyond the oral cavity, studies on bronchial epithelial cells from cystic fibrosis revealed that T2R14 contributes to calcium-mediated immune responses, promoting nitric oxide, interleukin, and human β-defensin 2 production, thereby enhancing airway epithelial defense against pathogens [89]. Consistently, specific *TAS2R* genotypes have been associated with higher expression of antimicrobial peptides and nitric oxide, and a lower risk of chronic rhinosinusitis [32]. Collectively, these findings, together with evidence of T2R roles in airway clearance [12,13,14,15,16,17], suggest that *TAS2R* variants may influence disease susceptibility by modulating airway defense mechanisms.

We identified three SNPs in *TAS2R1*, *TAS2R5*, and *TAS2R62P* associated with the SARS-CoV-2 antibody response. Growing evidence suggests that, in addition to their role in innate immunity, T2Rs regulate the adaptive immune response [79]. *TAS2Rs* are expressed in lymphocytes, with higher levels observed in younger adults compared to older adults [11,79]. Tran et al. reported significantly higher levels of T2R38 in activated lymphocytes compared to controls, as well as in central and effector memory cells compared to naïve cells [11]. Grassin-Delyle et al. demonstrated that T2R5 activation in human macrophages suppresses the secretion of pro-inflammatory cytokines [24]. Since these cytokines are crucial for B cell activation and proliferation, T2Rs may indirectly impact the antibody response to SARS-CoV-2 [11,24,90]. The SNPs identified to be associated with antibody seroconversion in this study include synonymous, 5′ UTR, and pseudogene variants. Although pseudogenes do not produce functional proteins, they are transcribed into RNA and contribute to gene expression regulation [29,30]. Additionally, synonymous variants may regulate post-transcriptional gene expression by affecting mRNA stability and translation speed [91,92].

The logistic regression results of this study demonstrated a significant association between respiratory diseases and SARS-CoV-2 confirmed or probable infection. This may be because probable cases, defined as those diagnosed by a physician, were more likely to present symptoms. Thus, respiratory disease appears to be associated with symptomatic or more severe COVID-19, which is consistent with previous research [93,94]. Interestingly, results from the UK Biobank Imputed Version 3 dataset, generated by the Neale Lab [95], support a connection between the same *TAS2R* variants reported here and non-COVID-19-related respiratory diseases: rs3851584 (G) in *TAS2R14* and respiratory disease symptoms (*p* < 0.001; effect size reported for T allele:−0.054); rs117458236 (T) in *TAS2R20* and pulmonary inflammation or edema (*p* = 0.02; effect size: 1.5); rs2234010 (A) in *TAS2R5 and* dependence on respirator (*p* = 0.001; effect size: 0.72). On the other hand, rs2234235 (G) in *TAS2R1*, identified in this study as a risk allele for nucleocapsid antibody response, was linked to a decreased risk of chronic airway obstruction in the UK Biobank data (*p* = 0.01; effect size: −0.097) [95].

In addition, consistent with previous research, we found a significant association between IMID and lower odds of spike antibody response (OR [95% CI]: 0.65 [0.49, 0.86]; *p* = 0.003), which is likely due to medication use and immune system dysregulation in IMID patients [28,48,96,97,98]. Patients with autoimmune diseases, such as rheumatoid arthritis, have approximately twice the risk of COVID-19, primarily due to glucocorticoid medication [49]. Our previous study on 21,991 CLSA participants showed an association between *TAS2R* genetic variants and temporomandibular symptoms, which can occur in rheumatoid arthritis involving the temporomandibular joint [43,99,100,101,102,103]. Nine of the fifteen variants linked to temporomandibular joint syndromes were located in *TAS2R20* [43]. A different SNP in the same gene was found to be associated with COVID-19 infection in the present study. Furthermore, the SNP rs3851584 in *TAS2R14*, which showed a borderline association with COVID-19 infection in this study, was also linked to temporomandibular joint symptoms in the previous study [43].

Subgroup analyses may clarify the interplay between *TAS2R* variants, COVID-19 outcomes, IMID, and respiratory disease. The rs2234235 (G) variant in *TAS2R1* was associated with higher odds of an anti-nucleocapsid response and lower odds of an anti-spike response (Table 4), despite showing no association with confirmed/probable infection in the overall cohort (Table 2). However, it was significantly associated with higher odds of confirmed/probable infection in individuals with respiratory disease or IMID. Additionally, although this variant was linked to reduced odds of anti-spike in the overall cohort, a subgroup analysis revealed a stronger effect in patients with respiratory disease or IMID. These findings suggest that the G allele of rs2234235 may impair antibody responses, potentially increasing susceptibility to SARS-CoV-2 infection in individuals at risk. This finding is further supported by GWAS results from the UK Biobank COVID-19 dataset, available through the GRASP portal [104], which revealed a significant association between the G allele of rs2234235 and severe COVID-19 outcomes in the European population (*p* = 0.001).

Self-reported diagnosis can be challenging to validate. A systematic review of systemic conditions in the CLSA found that certain conditions, such as respiratory disease, cardiovascular disease, and diabetes, could be accurately diagnosed using algorithms based on questionnaire data [56]. However, validating IMID diagnosis, including inflammatory bowel disease and musculoskeletal conditions such as rheumatoid arthritis, poses challenges, as necessary clinical variables are not included in the CLSA [56]. To improve the accuracy of IMID diagnosis, we incorporated specific medications used for these conditions into our algorithms (Table S1). Another study on the CLSA cohort found that incorporating medication data into chronic disease assessment can resolve over 93% of inconsistent responses [57]. Additionally, Oremus et al. demonstrated that an ischemic heart disease algorithm could achieve optimal sensitivity and specificity using self-reported data alone, without ECG confirmation [58]. The prevalence estimates for systemic conditions in this study were comparable to those reported by the Canadian Chronic Disease Surveillance System (CCDSS) for individuals aged 45 to 84 years and other CLSA studies [59,65,68,70,71].

This study has some limitations. Only individuals of European ancestry were included, which limits the extension of the findings to other populations. Furthermore, the CLSA cohort consists of individuals who volunteered to participate. This may result in selection bias and the underrepresentation of populations at higher risk for COVID-19, including those with lower socioeconomic status, limited access to healthcare, minorities, and individuals with comorbidities. Further studies in more diverse populations are needed to confirm the generalizability of these results. Additionally, only 16% of the Questionnaire cohort reported a nucleic acid amplification test for COVID-19, resulting in a small sample size for the confirmed infection outcome. Although a larger population was included in the confirmed/probable category, some individuals may not have been examined by a physician or may have been asymptomatic, resulting in data that remained skewed towards individuals without COVID-19 infection. Although we controlled for multiple potential confounders, environmental factors and genetic variants in other genes were not taken into account. Furthermore, as this study focused on COVID-19, it remains unclear whether the identified variants are also associated with other viral or bacterial infections. The potential impact of medications known to affect serological responses, particularly B-cell-targeted therapies often used in IMIDs, could not be accounted for in this study due to the small numbers of individuals on these medications. All serological samples were submitted over 2 weeks following any reported COVID-19 test; thus, the risk of false-negative nucleocapsid antibody testing is low. However, 4% of serological samples were submitted within 2 weeks of the first vaccine dose, and of these 76% (representing 3% of the total cohort) tested negative for spike antibody and thus were possible false negatives. Additionally, infection and vaccine-mediated immunogenicity are known to decrease over time, which may have impacted the serological data, resulting in low rates of serologically defined infection or vaccine response. Further research is needed to investigate the role of T2Rs in B lymphocyte activation and antibody production, as well as to explore the functional impact of the associated variants on T2Rs and their potential causal relationship with SARS-CoV-2 and other airborne pathogens. Importantly, the identified associations of *TAS2R* variants with infection or serology are preliminary and require validation in separate, larger cohorts with higher infection prevalence.

## 5. Conclusions

This study highlights *TAS2R* variants as potential contributors to COVID-19 infection and immune response. Several *TAS2R* variants were found to increase the risk of infection while reducing antibody seroconversion following vaccination. Identifying specific variants linked to disease risk could help in evaluating prognosis, guiding treatment planning, and prioritizing vaccination for individuals carrying high-risk genotypes. Given that COVID-19 continues to generate significant healthcare burdens, particularly for older adults and individuals with chronic diseases, these data may inform vaccination strategies for COVID-19 and provide insights into early innate immune responses against emerging and endemic airborne pathogens.

## Figures and Tables

**Table 1:** Population characteristics of the study cohorts.

Characteristic	COVID-19 Questionnaire (N = 14,073) ^1^	COVID-19 Antibody (N = 8313) ^1^
Sex		
Male	6831 (49%)	4133 (50%)
Female	7242 (51%)	4180 (50%)
Age	67 (61, 75)	67 (60, 75)
BMI	26.9 (24.1, 30.5)	27.1 (24.3, 30.5)
Marital partner status		
Single/never married	1113 (7.9%)	617 (7.4%)
Married/in a common-law relationship	9728 (69%)	5816 (70%)
Widowed/divorced/separated	3201 (23.2%)	1857 (22.3%)
Dwelling ownership		
Own	11,824 (85%)	7075 (86%)
Rent	2064 (15%)	1125 (14%)
Smoking	857 (6.2)	401 (4.9%)
Alcohol consumption		
Never	1843 (13%)	1039 (13%)
Less than 4 times per month	3725 (27%)	2182 (26%)
1–3 times per week	4405 (31%)	2635 (32%)
More than 3 times per week	4080 (29%)	2445 (29%)
Number of household residents	2 (2, 2)	2 (2, 2)
Post-secondary education	12,307 (88%)	7321 (88%)
Diabetes ^2^	3099 (22%)	1828 (22%)
Cardiovascular disease ^3^	7229 (51%)	4204 (51%)
Respiratory disease ^4^	2890 (21%)	1723 (21%)
Immune-mediated inflammatory disease ^5^	829 (5.9%)	491 (5.9%)
Tested for COVID-19 ^6^	2299 (16%)	2510 (31%)
Self-reported positive test for COVID-19	45	78

^1^ n (%); median (IQR); percentages are calculated based on respondents, excluding missing data. ^2^ Diabetes, borderline diabetes, or high blood sugar. ^3^ Hypertension, peripheral arterial disease or poor circulation in limbs, heart disease, heart attack or myocardial infarction, angina, or history of stroke or CVA. ^4^ Asthma, emphysema, chronic bronchitis, chronic obstructive pulmonary disease (COPD), or chronic pulmonary changes due to smoking. ^5^ Rheumatoid arthritis or inflammatory bowel disease.^6^ Self-reported nucleic acid amplification test.

**Table 2:** Association between *TAS2R* variants and COVID-19 infection in the CLSA COVID-19 Questionnaire cohort with available genetic data.

Characteristic	Confirmed/Probable COVID-19 (N = 13,536) ^1^			Confirmed COVID-19 (N = 2204) ^2^		
	Controls (N = 13,389)	Cases (N = 147)		Controls (N = 2161)		Cases (N = 43)
	OR ^3^	95% CI ^3^	*p*-Value	OR	95% CI	*p*-Value
Age	0.97	0.95, 0.99	<0.001	1.02	0.99, 1.06	0.24
Sex (female)	1.80	1.28, 2.58	<0.001	1.45	0.76, 2.87	0.27
Smoking	0.75	0.33, 1.45	0.44	NA ^6^	NA	NA
Dwelling area (urban core)	1.48	0.91, 2.56	0.14	1.37	0.57, 4.09	0.52
Dwelling ownership (rent)	1.66	1.08, 2.49	0.017	0.96	0.42, 2.01	0.91
Post-secondary education	1.44	0.83, 2.71	0.22	1.25	0.53, 3.49	0.64
Diabetes	1.40	0.94, 2.03	0.088	1.05	0.47, 2.14	0.91
Respiratory disease	1.78	1.24, 2.52	0.001	1.15	0.55, 2.25	0.69
Cardiovascular disease	0.84	0.59, 1.20	0.35	0.89	0.46, 1.73	0.73
IMID ^4^	1.33	0.69, 2.34	0.35	2.62	0.95, 6.18	0.041
rs2234235, *TAS2R1* (A/G)	1.48	0.80, 2.52	0.18	1.01	0.24, 2.78	0.99
rs2234009, *TAS2R5* (C/T)	1.09	0.49, 2.07	0.81	1.39	0.33, 4.01	0.60
rs2234010, *TAS2R5* (G/A)	0.89	0.40, 1.70	0.74	0.81	0.13, 2.81	0.78
rs1726866, *TAS2R38* (A/G)	1.09	0.86, 1.38	0.47	1.06	0.68, 1.66	0.79
rs34039200, *TAS2R62P* (G/A)	1.17	0.89, 1.52	0.26	1.41	0.86, 2.28	0.16
rs3851584, *TAS2R14* ^5^ (T/G)	1.26	0.99, 1.60	0.058	1.18	0.75, 1.85	0.47
rs77837442, *TAS2R19* (C/T)	0.75	0.18, 2.02	0.63	NA ^6^	NA	NA
rs117458236, *TAS2R20* (C/T)	1.95	0.98, 3.51	0.039	2.40	0.55, 7.35	0.17

^1^ Defined as a “yes” response to either of the following questions: “Have you had a positive test result?” or “Have you been told by a healthcare provider that you have COVID-19, but you did NOT have a test to confirm this?”. ^2^ Defined as a “yes” response to the question, “Have you had a positive test result?”. ^3^ Logistic regression; OR = Odds Ratio; CI = Confidence Interval. ^4^ IMID = Immune-mediated inflammatory disease. ^5^ In linkage disequilibrium with rs10772397 in TAS2R50 (r2 = 0.83) and rs1015443 and rs1015442 in *TAS2R13* (r2 > 0.99). ^6^ Not included in logistic regression.

**Table 3:** COVID-19 vaccination history and antibody response in the COVID-19 Antibody Study cohort with available genetic data.

Characteristic	Number (N = 8313)
Number of doses of COVID-19 vaccine ^1,2^	
0	1438
1	4267
2 or more	869
Missing	1739
Vaccine type ^3^	
Pfizer BioNTech	3901
Moderna	600
AstraZeneca	695
Other type	28
Missing	55
Nucleocapsid antibody response	370
Spike antibody response	3773
Antibody result interpretation	
Prior SARS-CoV-2 infection ^4^	151
Prior SARS-CoV-2 infection and/or vaccination ^5^	3554
Prior SARS-CoV-2 infection OR infection and vaccination ^6^	219
No antibodies detected	3757
Missing	632

^1^ “Have you received at least one dose of a COVID-19 vaccine?”. ^2^ “How many doses of COVID-19 vaccine have you received so far?”. ^3^ “Which vaccine did you receive?”. ^4^ Positive nucleocapsid antibody and negative spike antibody. ^5^ Negative nucleocapsid antibody and positive spike antibody. ^6^ Positive nucleocapsid antibody and positive spike antibody.

**Table 4:** Association between *TAS2R* variants and SARS-CoV-2 seroconversion in the CLSA COVID-19 Antibody cohort with available genetic data.

Characteristic	Nucleocapsid Antibody (N = 7475)			Spike Antibody (N = 7475)		
	Controls (N = 7115)	Cases (N = 360)		Controls (N = 3818)		Cases (N = 3657)
	OR ^1^	95% CI ^1^	*p*-Value	OR	95% CI	*p*-Value
Age	0.99	0.98, 1.01	0.30	1.00	0.99, 1.01	0.81
Sex (female)	0.87	0.70, 1.08	0.22	1.25	1.08, 1.43	0.002
Smoking	0.84	0.47, 1.39	0.54	0.94	0.68, 1.33	0.74
Dwelling ownership (rent)	0.94	0.67, 1.30	0.73	0.93	0.76, 1.15	0.50
Number of household residents	1.05	0.95, 1.12	0.27	1.05	0.98, 1.12	0.21
Number of vaccine doses	0.98	0.82, 1.16	0.79	90.2	72.6, 114	<0.001
Post-secondary education	0.84	0.61, 1.17	0.29	1.16	0.93, 1.43	0.19
Diabetes	0.94	0.71, 1.23	0.67	0.89	0.75, 1.06	0.19
Respiratory disease	1.08	0.82, 1.40	0.57	0.93	0.79, 1.10	0.40
Cardiovascular disease	0.96	0.76, 1.21	0.72	0.90	0.78, 1.04	0.16
IMID ^2^	1.16	0.73, 1.76	0.50	0.65	0.49, 0.86	0.003
rs2234235, *TAS2R1* (A/G)	1.55	1.06, 2.20	0.018	0.74	0.57, 0.98	0.033
rs2234009, *TAS2R5* (C/T)	1.01	0.62, 1.56	0.96	1.33	0.99, 1.80	0.064
rs2234010, *TAS2R5* (G/A)	1.56	1.08, 2.19	0.014	1.26	0.95, 1.69	0.11
rs1726866, *TAS2R38* (A/G)	0.89	0.76, 1.03	0.13	1.04	0.94, 1.14	0.46
rs34039200, *TAS2R62P* (G/A)	0.99	0.82, 1.19	0.93	0.86	0.77, 0.97	0.013
rs3851584, *TAS2R14* (T/G)	0.96	0.82, 1.12	0.63	1.02	0.93, 1.13	0.65
rs77837442, *TAS2R19* (C/T)	0.45	0.16, 0.99	0.080	0.91	0.63, 1.34	0.63
rs117458236, *TAS2R20* (C/T)	0.99	0.53, 1.68	0.96	0.94	0.67, 1.33	0.73

^1^ Logistic regression; OR = Odds Ratio; CI = Confidence Interval. ^2^ IMID = immune-mediated inflammatory disease.

**Table 5:** Association between *TAS2R* variants and SARS-CoV-2 spike antibody in individuals who received at least one dose of vaccine and did not have detectable nucleocapsid antibody (infection) (N = 4404).

Characteristic	Spike Antibody		
	OR ^1^	95% CI ^1^	*p*-Value
Age	1.00	0.99, 1.01	0.72
Sex	1.31	1.13, 1.53	<0.001
Smoking	0.92	0.65, 1.34	0.66
Dwelling ownership (rent)	0.90	0.73, 1.13	0.37
Number of household residents	1.06	0.98, 1.16	0.17
Number doses	8.26	5.71, 12.5	<0.001
Vaccine type			
Moderna	2.33	1.34, 4.05	0.003
Pfizer BioNTech	1.52	0.91, 2.51	0.11
AstraZeneca	1.25	0.73, 2.14	0.42
Post-secondary education	1.14	0.90, 1.43	0.28
Diabetes	0.85	0.71, 1.02	0.078
Respiratory disease	0.87	0.72, 1.04	0.12
Cardiovascular disease	0.87	0.74, 1.01	0.074
IMID ^2^	0.62	0.46, 0.83	0.001
rs2234235, *TAS2R1* (A/G)	0.71	0.54, 0.95	0.021
rs2234009, *TAS2R5* (C/T)	1.26	0.92, 1.75	0.17
rs2234010, *TAS2R5* (G/A)	1.17	0.86, 1.62	0.34
rs1726866, *TAS2R38* (A/G)	1.02	0.92, 1.14	0.69
rs34039200, *TAS2R62P* (G/A)	0.86	0.76, 0.97	0.017
rs3851584, *TAS2R14* (T/G)	1.05	0.95, 1.17	0.34
rs77837442, *TAS2R19* (C/T)	0.95	0.64, 1.43	0.79
rs117458236, *TAS2R20* (C/T)	0.88	0.62, 1.27	0.47

^1^ Logistic regression; OR = Odds Ratio; CI = Confidence Interval. ^2^ IMID = immune-mediated inflammatory disease.

## Data Availability

This study has been conducted using the CLSA Genome-wide Genetic Data Release (version 3), Comprehensive Baseline Dataset (version v7.0), Comprehensive Follow-up 2 Dataset (v1.1), COVID-19 Questionnaire Study, and COVID-19 Seroprevalence (Antibody) Study. Data are available from the Canadian Longitudinal Study on Aging (https://www.clsa-elcv.ca/) for researchers who meet the criteria for access to de-identified CLSA data. The source code of this article has been deposited at https://github.com/mshafizadeh/TAS2R_COVID19 (accessed on 7 March 2025) and is publicly available as of the date of publication.

## Supplementary Materials

The following supporting information can be downloaded at https://www.mdpi.com/article/10.3390/biomedicines13102473/s1. Figure S1: Algorithms for defining chronic conditions in CLSA. Follow-up 2 data was used to determine medication use, based on responses to the question: “Do you use any medications for …?” For rheumatoid arthritis and inflammatory bowel disease, medication data (listed in Table S1) was obtained from the Baseline dataset, as this information was not available in the Follow-up 2 data; Table S1: List of medications for rheumatoid arthritis and inflammatory bowel disease, excluding steroids. Drug identification numbers (DINs) were retrieved from the Health Canada Drug Product Database; Table S2: Variables initially extracted for logistic regression; Figure S2: Correlation plots of covariates. (A) COVID-19 cohort (n = 14,073). (B) COVID-19 Antibody cohort (n = 8313). IMID: immune-mediated inflammatory disease; Table S3: *TAS2R* variants with differing allele frequencies by COVID-19-related phenotypes. Only variants with *P* < 0.05 are presented (chi-squared test); Table S4: Association between *TAS2R* variants and confirmed/probable COVID-19 infection in patients with chronic medical conditions and in corresponding control groups, defined as individuals without the specific condition under analysis; Table S5: Association between *TAS2R* variants and SARS-CoV-2 nucleocapsid antibody in patients with chronic medical conditions and in corresponding control groups, defined as individuals without the specific condition under analysis; Table S6: Association between *TAS2R* variants and SARS-CoV-2 spike antibody in patients with chronic medical conditions and in corresponding control groups, defined as individuals without the specific condition under analysis; Figure S3: Predicted mRNA secondary structures and base pair probabilities calculated by RNAsnp. In each matrix, the upper triangle represents base pair probabilities for the wild-type sequence, and the lower triangle represents probabilities for the mutant sequence. Red and green dots indicate base pairs whose probabilities differ between the two alleles. The results reveal structural changes in mRNA for the variants, with the rs2234010 in *TAS2R5* predicted to have the most notable impact.

## Abbreviations

The following abbreviations are used in this manuscript:

CLSA	Canadian Longitudinal Study on Aging
COVID-19	Coronavirus disease 2019
CI	(95% confidence interval)
IMID	Immune-mediated inflammatory disease
OR	odds ratio
PCA	principal component analysis
SARS-CoV-2	severe acute respiratory syndrome coronavirus 2
SNPs	single nucleotide polymorphisms
T2R	bitter taste receptor
*TAS2R*	bitter taste receptor gene
