# Supplementary material for: Genetic Variations in Bitter Taste Receptors and COVID-19 in the Canadian Longitudinal Study on Aging
Source: Biomedicines. 2025 Oct 11;13(10):2473. doi: 10.3390/biomedicines13102473 (PMC12562077; doi:10.3390/biomedicines13102473)
Supplement: Supplementary file 1 [file biomedicines-13-02473-s001.zip › biomedicines-3817516-supplementary.pdf]

Figure S1. Algorithms for defining chronic conditions in CLSA. Follow-up 2 data was used to determine medication use, based on responses to the question: "Do you use any medications for …?" For rheumatoid arthritis and inflammatory bowel disease, medication data (listed in Table S1) was obtained from the Baseline dataset, as this information was not available in the Follow-up 2 data.

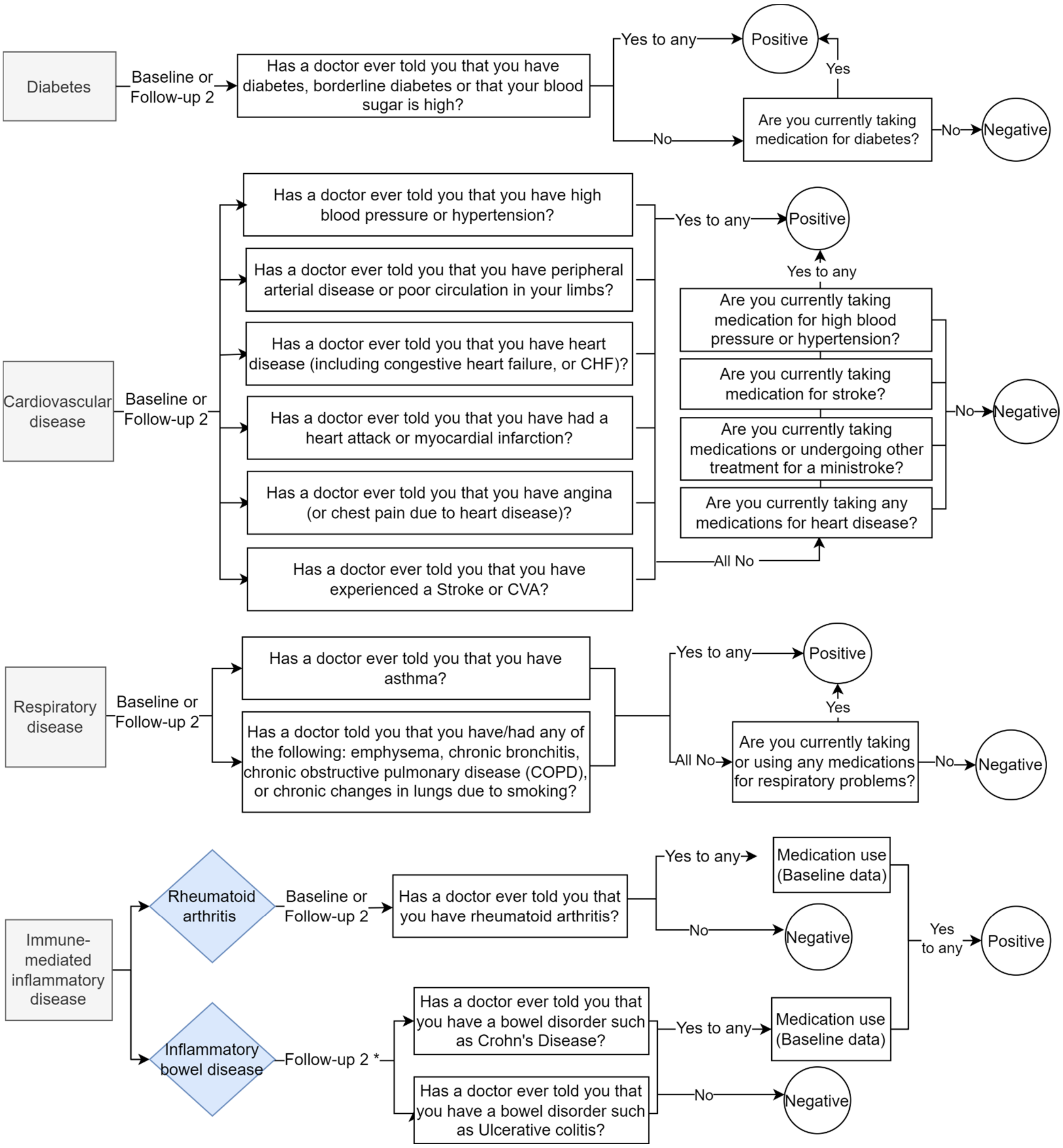

Table S1. List of medications for rheumatoid arthritis and inflammatory bowel disease, excluding steroids. Drug identification numbers (DINs) were retrieved from the Health Canada Drug Product Database.

Disease	Immunomodulators	Immunosuppressants	Biologics and small molecules
Rheumatoid arthritis	Cyclosporin<br>Hydroxychloroquine<br>Minocycline<br>Sulfasalazine	Azathioprine<br>Cyclophosphamide<br>Leflunomide<br>Methotrexate<br>Mycophenolate	Infliximab<br>Adalimumab<br>Etanercept<br>Certolizumab<br>Golimumab<br>Rituximab<br>Abatacept<br>Tocilizumab<br>Tofacitinib<br>Upadacitinib<br>Baricitinib
Inflammatory bowel disease	Mesalazine or mesalamine (5-ASA, Mezavant, Salofalk, Asacol, Pentasa, Mesasal, Mezera, Octasa)<br><br>Thiopurines (Thioguanine)<br><br>Sulfasalazine<br>Dipentum	Azathioprine<br>6-mercaptopurine<br>Methotrexate	Infliximab<br>Adalimumab<br>Golimumab<br>Ustekinumab<br>Vedolizumab

Table S2. Variables initially extracted for logistic regression.

Variable	Dataset	Question	Response values/categories
Sex	Genetic data	-	Female/male (chromosomal)
ePC1-10 [1]	Genetic data	-	Continuous variable
Dwelling area	COVID-19	-	Dichotomized: urban core, rural/not urban core
Number of household residents	Antibody	How many people, including yourself, live in your residence? (sleep there at least 3 nights per week)	Integer
Smoking status	COVID-19 and Antibody	At the present time, do you smoke cigarettes daily, occasionally, or not at all?	Dichotomized: daily/occasionally, not at all
Education	Baseline	What is your highest level of education?	Dichotomized: with or without post-secondary education/graduation
Province of residence	Follow-up 2	-	Newfoundland and Labrador, Prince Edward Island, Nova Scotia, New Brunswick, Quebec, Ontario, Manitoba, Saskatchewan, Alberta, British Columbia, Yukon, Northwest Territories, Nunavut
Alcohol consumption	Follow-up 2	About how often during the past 12 months did you drink alcohol?	Never, 2-3 times per month or less, 1-3 times per week, more than 3 times per week
Systemic conditions	Follow-up 2 and Baseline	Based on the case definitions explained in Figure 2S	Diabetes, cardiovascular disease, respiratory disease, immune-mediated inflammatory disease
Age (years)	Follow-up	-	Integer
Body mass index (BMI)	Follow-up 2	-	Decimal (pregnant women were excluded)
Marital/partner status	Follow-up 2	What is your current marital/partner status?	Single (never married/lived with a partner), married or in a common-law relationship, widowed, divorced, separated
Retirement status	Follow-up 2	At this time, do you consider yourself to be completely retired, partly retired, or not retired?	Completely retired, partly retired, not retired, never had a paid job
Dwelling status	Follow-up 2	Do you or your partner own or rent your dwelling?	Own, rent, other
Received vaccine	Antibody	Have you received at least one dose of a COVID-19 vaccine?	Yes, No
Number of vaccines received	Antibody	How many doses of COVID-19 vaccine have you received so far?	Integer
Type of vaccine	Antibody	Which vaccine did you receive?	Pfizer and BioNTech mRNA vaccine, Moderna mRNA vaccine, AstraZeneca Oxford, Other

[1] Top 10 components of the principal component analysis (PCA) from the CLSA European ancestry population.

Figure S2. Correlation plots of covariates. **(A)** COVID-19 cohort (n=14,073). **(B)** COVID-19 Antibody cohort (n=8,313). IMID: immune-mediated inflammatory disease.

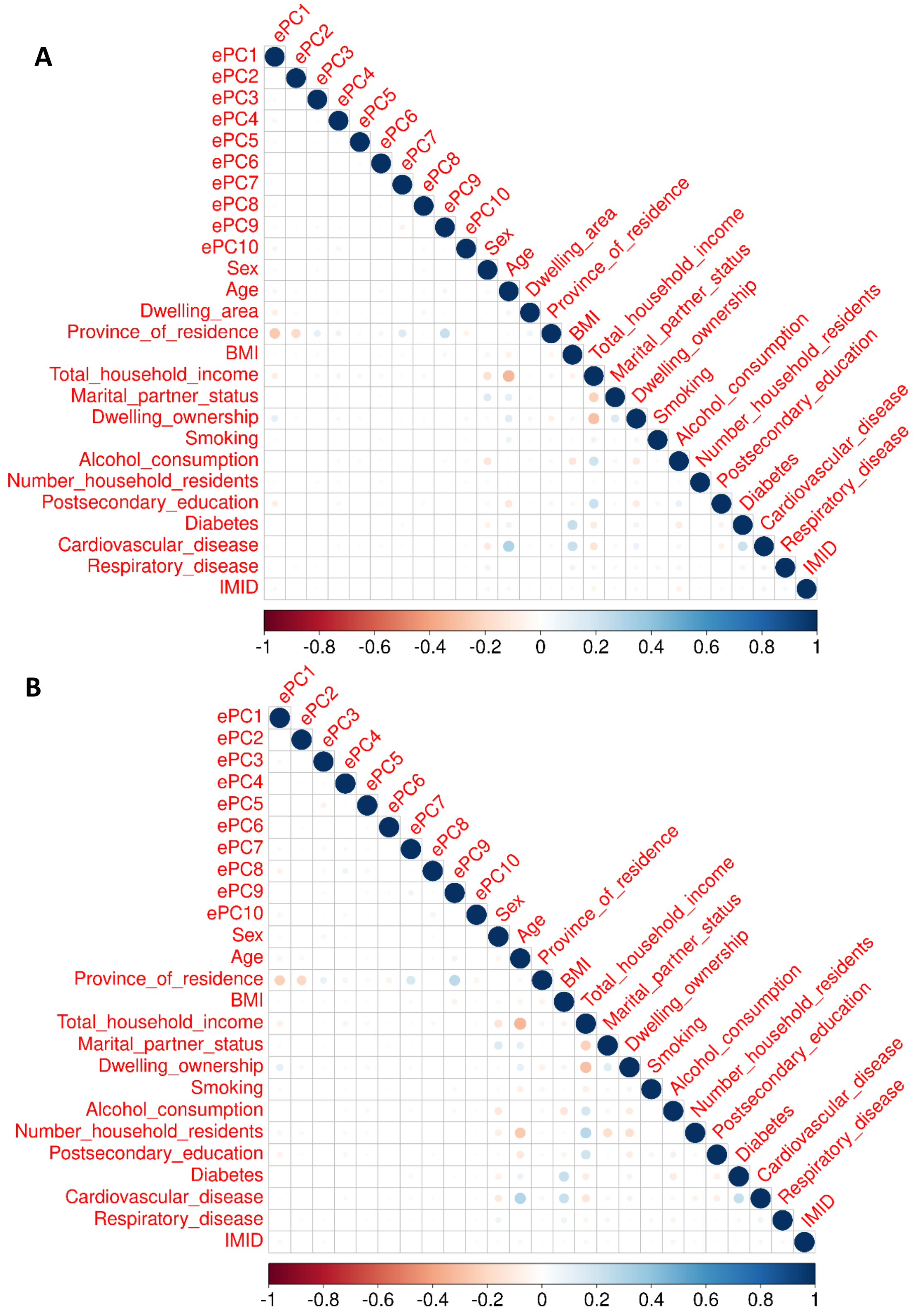

Table S3. *TAS2R* variants with differing allele frequencies by COVID-19-related phenotypes. Only variants with $P < 0.05$ are presented (chi-squared test).

Phenotype	rs ID	Gene	Allele[1]	OR (95% CI)[2]	Consequence	P-value	Adjusted P-value
Confirmed or probable infection	rs117458236	*TAS2R20*	T	2.13 (1.19, 3.82)	3′ UTR[3]	0.009	0.072
	rs3851584	*TAS2R14*	G	1.30 (1.03, 1.62)	3′ UTR	0.024	0.192
	rs1548803	*TAS2R8*	C	1.29 (1.03, 1.62)	Synonymous 183	0.028	0.224
	rs184006834	*TAS2R67p*	A	1.64 (1.05, 2.57)	Downstream (SMIM10L1)	0.028	0.224
	rs1015443	*TAS2R13*	T	1.28 (1.02, 1.61)	Missense (Ser 259 Asn)	0.030	0.240
	rs1015442	*TAS2R13*	T	1.28 (1.02, 1.61)	3′ UTR	0.031	0.248
	rs10772397	*TAS2R50*	C	1.28 (1.02, 1.61)	Synonymous 259	0.031	0.248
	rs3741845	*TAS2R9*	A	1.27 (1.01, 1.59)	Missense (Ala 187 Val)	0.038	0.304
Nucleocapsid antibody	rs2234010	*TAS2R5*	A	1.60 (1.13, 2.27)	5′ UTR	0.008	0.064
	rs2234235	*TAS2R1*	G	1.57 (1.10, 2.24)	Synonymous	0.013	0.104
	rs10246939	*TAS2R38*	C	0.85 (0.73, 0.99)	Missense (Ile 296 Val)	0.034	0.272
	rs1726866	*TAS2R38*	G	0.85 (0.74, 0.99)	Missense (Val 262 Ala)	0.040	0.320
	rs713598	*TAS2R38*	G	0.85 (0.73, 0.99)	Missense (Ala 49 Pro)	0.040	0.320
	rs77837442	*TAS2R19*	T	0.44 (0.18, 1.06)	Stop-gained (Trp 295)	0.059	0.472
Spike antibody	rs34039200	*TAS2R62p*	A	0.92 (0.85, 0.99)	Pseudogene	0.026	0.208
	rs2234009	*TAS2R5*	T	1.22 (1.00, 1.48)	5′ UTR	0.050	0.400

[1] Effect Allele.
[2] OR = Odds Ratio, CI = Confidence Interval.
[3] UTR = Untranslated region.

Table S4. Association between *TAS2R* variants and confirmed/probable COVID-19 infection in patients with chronic medical conditions and in corresponding control groups, defined as individuals without the specific condition under analysis.

**Characteristic**	**Diabetes**				**Cardiovascular disease**			
	**Controls (N=10,560)**		**Patients (N=2,976)**		**Controls (N=6,613)**		**Patients (N=6,923)**	
	**OR (95% CI)[1]**	**p-value**	**OR (95% CI)[1]**	**p-value**	**OR (95% CI)[1]**	**p-value**	**OR (95% CI)[1]**	**p-value**
**Age**	**0.97 (0.95, 0.99)**	**0.011**	**0.95 (0.91, 0.99)**	**0.018**	**0.97 (0.94, 0.99)**	**0.014**	**0.97 (0.94, 1.00)**	**0.023**
**Sex (female)**	**1.89 (1.25, 2.90)**	**0.003**	1.69 (0.88, 3.31)	0.12	**2.20 (1.35, 3.76)**	**0.002**	1.49 (0.91, 2.47)	0.11
Smoking	0.39 (0.09, 1.04)	0.11	1.99 (0.66, 4.92)	0.17	0.64 (0.19, 1.56)	0.39	0.93 (0.28, 2.32)	0.90
Dwelling area (urban core)	1.60 (0.90, 3.09)	0.13	1.15 (0.48, 3.43)	0.77	1.53 (0.81, 3.20)	0.22	1.48 (0.71, 3.60)	0.34
**Dwelling (rent)**	1.62 (0.95, 2.64)	0.063	1.98 (0.91, 4.04)	0.071	**1.88 (1.02, 3.26)**	**0.032**	1.48 (0.78, 2.64)	0.21
**Post-secondary education**	1.42 (0.74, 3.06)	0.33	1.62 (0.62, 5.53)	0.38	0.81 (0.42, 1.71)	0.55	**3.61 (1.32, 14.9)**	**0.032**
Diabetes	-	-	-	-	1.20 (0.61, 2.16)	0.56	1.55 (0.93, 2.54)	0.088
Cardiovascular disease	0.77 (0.50, 1.16)	0.22	1.11 (0.55, 2.35)	0.78	-	-	-	-
**Respiratory disease**	**2.16 (1.42, 3.22)**	**<0.001**	1.00 (0.47, 2.00)	>0.99	**1.81 (1.09, 2.91)**	**0.018**	**1.72 (1.01, 2.85)**	**0.039**
IMID[2]	1.10 (0.46, 2.25)	0.80	1.72 (0.57, 4.23)	0.28	1.19 (0.41, 2.72)	0.72	1.39 (0.57, 2.90)	0.42
**rs2234235, *TAS2R1***	**2.08 (1.11, 3.57)**	**0.013**	0.00 (0.00, 15,096,353)	0.98	1.88 (0.87, 3.57)	0.078	1.01 (0.31, 2.45)	0.98
rs2234009, *TAS2R5*	1.41 (0.59, 2.86)	0.38	0.42 (0.02, 1.92)	0.39	0.77 (0.19, 2.07)	0.65	1.43 (0.51, 3.16)	0.44
rs2234010, *TAS2R5*	0.74 (0.26, 1.65)	0.52	1.21 (0.29, 3.37)	0.76	1.22 (0.47, 2.60)	0.64	0.49 (0.08, 1.56)	0.32
rs1726866, *TAS2R38*	1.15 (0.88, 1.52)	0.31	0.93 (0.58, 1.47)	0.75	1.19 (0.86, 1.64)	0.29	0.97 (0.68, 1.37)	0.85
rs34039200, *TAS2R62P*	1.22 (0.88, 1.65)	0.22	1.05 (0.59, 1.78)	0.87	1.23 (0.85, 1.74)	0.27	1.08 (0.71, 1.62)	0.70
**rs3851584, *TAS2R14***	1.28 (0.97, 1.68)	0.086	1.14 (0.71, 1.80)	0.59	**1.38 (1.00, 1.90)**	**0.050**	1.13 (0.79, 1.60)	0.52
rs77837442, *TAS2R19*	0.66 (0.11, 2.11)	0.56	0.97 (0.05, 4.87)	0.98	1.39 (0.33, 3.82)	0.59	0.00 (0.00, 50.4)	0.98
**rs117458236, *TAS2R20***	1.65 (0.68, 3.38)	0.21	**3.27 (0.93, 8.91)**	**0.035**	1.29 (0.38, 3.22)	0.63	**2.69 (1.10, 5.65)**	**0.016**

[1]Logistic regression; OR = Odds Ratio; CI = Confidence Interval.

[2]IMID = immune-mediated inflammatory disease.

Table S4 (Continued). Association between *TAS2R* variants and confirmed/probable COVID-19 infection in patients with chronic medical conditions and in corresponding control groups, defined as individuals without the specific condition under analysis.

**Characteristic**	**Respiratory disease**				**Immune-mediated inflammatory disease**			
	**Controls (N=10,757)**		**Patients (N=2,779)**		**Controls (N=12,739)**		**Patients (N=797)**	
	**OR (95% CI)[1]**	**p-value**	**OR (95% CI)[1]**	**p-value**	**OR (95% CI)[1]**	**p-value**	**OR (95% CI)[1]**	**p-value**
**Age**	**0.97 (0.94, 0.99)**	**0.005**	**0.96 (0.93, 1.00)**	**0.034**	**0.96 (0.94, 0.98)**	**<0.001**	1.00 (0.93, 1.07)	0.95
**Sex (female)**	**1.83 (1.21, 2.82)**	**0.005**	1.79 (0.96, 3.52)	0.075	**1.73 (1.21, 2.50)**	**0.003**	4.15 (0.97, 29.4)	0.087
Smoking	0.46 (0.11, 1.25)	0.19	1.32 (0.44, 3.15)	0.57	0.82 (0.36, 1.59)	0.59	0.00 (-, -)	>0.99
Dwelling area (urban core)	1.31 (0.75, 2.48)	0.38	2.12 (0.84, 7.18)	0.16	1.56 (0.93, 2.80)	0.11	0.85 (0.19, 6.28)	0.85
**Dwelling rent**	1.62 (0.94, 2.68)	0.070	1.74 (0.83, 3.42)	0.12	**1.78 (1.14, 2.71)**	**0.009**	0.82 (0.11, 3.75)	0.82
Post-secondary education	1.91 (0.94, 4.61)	0.10	0.94 (0.41, 2.55)	0.89	1.52 (0.85, 3.03)	0.19	1.23 (0.28, 8.95)	0.81
**Diabetes**	**1.73 (1.08, 2.71)**	**0.020**	0.90 (0.43, 1.76)	0.77	1.32 (0.87, 1.96)	0.19	3.21 (0.82, 12.5)	0.086
Cardiovascular disease	0.83 (0.53, 1.28)	0.40	0.91 (0.49, 1.68)	0.75	0.84 (0.58, 1.21)	0.35	0.80 (0.20, 3.41)	0.76
**Respiratory disease**	-	-	-	-	**1.93 (1.33, 2.77)**	**<0.001**	0.77 (0.16, 2.79)	0.71
IMID[2]	1.83 (0.85, 3.49)	0.092	0.67 (0.16, 1.88)	0.50	-	**-**	-	-
**rs2234235, *TAS2R1***	0.99 (0.39, 2.08)	0.99	**2.65 (1.08, 5.61)**	**0.018**	1.24 (0.61, 2.24)	0.52	**7.39 (1.36, 33.8)**	**0.012**
rs2234009, *TAS2R5*	0.72 (0.22, 1.72)	0.52	2.22 (0.67, 5.57)	0.13	1.19 (0.53, 2.27)	0.64	0.00 (-, -)	>0.99
rs2234010, *TAS2R5*	0.88 (0.34, 1.83)	0.75	0.86 (0.14, 2.90)	0.84	0.85 (0.36, 1.70)	0.69	1.03 (0.05, 6.89)	0.98
**rs1726866, *TAS2R38***	0.86 (0.64, 1.14)	0.30	**1.86 (1.22, 2.88)**	**0.004**	1.10 (0.86, 1.40)	0.45	0.94 (0.39, 2.25)	0.89
rs34039200, *TAS2R62P*	1.14 (0.81, 1.57)	0.43	1.22 (0.74, 1.94)	0.43	1.17 (0.87, 1.54)	0.29	1.18 (0.38, 3.21)	0.75
**rs3851584, *TAS2R14***	1.22 (0.91, 1.63)	0.19	1.36 (0.89, 2.08)	0.15	**1.30 (1.01, 1.67)**	**0.038**	1.04 (0.42, 2.54)	0.92
rs77837442, *TAS2R19*	0.75 (0.12, 2.42)	0.70	0.67 (0.04, 3.23)	0.69	0.82 (0.20, 2.20)	0.74	0.00 (-, -)	>0.99
**rs117458236, *TAS2R20***	2.09 (0.92, 4.13)	0.052	1.81 (0.42, 5.34)	0.34	**2.15 (1.08, 3.88)**	**0.018**	0.00 (-, -)	>0.99

[1]Logistic regression; OR = Odds Ratio; CI = Confidence Interval.
[2]IMID = immune-mediated inflammatory disease.

Table S5. Association between *TAS2R* variants and SARS-CoV-2 nucleocapsid antibody in patients with chronic medical conditions and in corresponding control groups, defined as individuals without the specific condition under analysis.

**Characteristic**	**Diabetes**				**Cardiovascular disease**			
	**Controls (N=5,875)**		**Patients (N=1,600)**		**Controls (N=3,759)**		**Patients (N=3,716)**	
	**OR (95% CI)[1]**	**p-value**	**OR (95% CI)[1]**	**p-value**	**OR (95% CI)[1]**	**p-value**	**OR (95% CI)[1]**	**p-value**
**Age**	1.00 (0.99, 1.02)	0.83	**0.96 (0.93, 0.99)**	**0.007**	0.99 (0.97, 1.01)	0.37	1.0 (0.98, 1.01)	0.56
Sex (female)	0.87 (0.68, 1.11)	0.26	0.80 (0.48, 1.31)	0.38	0.86 (0.64, 1.16)	0.32	0.87 (0.63, 1.20)	0.40
Smoking	0.95 (0.51, 1.63)	0.87	0.45 (0.07, 1.56)	0.29	0.99 (0.46, 1.89)	0.98	0.76 (0.29, 1.62)	0.52
**Dwelling rent**	0.90 (0.60, 1.32)	0.61	1.08 (0.53, 2.04)	0.81	**0.53 (0.27, 0.94)**	**0.045**	1.28 (0.83, 1.91)	0.25
Number of household residents	1.05 (0.95, 1.13)	0.28	1.04 (0.80, 1.25)	0.73	1.06 (0.94, 1.16)	0.25	1.02 (0.87, 1.14)	0.75
Number of vaccine doses	1.00 (0.83, 1.21)	>0.99	0.88 (0.60, 1.29)	0.53	1.02 (0.81, 1.29)	0.85	0.93 (0.72, 1.19)	0.58
Post-secondary education	0.88 (0.61, 1.32)	0.53	0.66 (0.36, 1.28)	0.20	0.85 (0.53, 1.47)	0.54	0.84 (0.55, 1.31)	0.41
Diabetes	-	-	-	-	0.96 (0.59, 1.50)	0.87	0.93 (0.66, 1.29)	0.67
Cardiovascular disease	0.92 (0.71, 1.19)	0.52	1.08 (0.64, 1.87)	0.79	-	-	-	-
Respiratory disease	1.00 (0.73, 1.35)	0.98	1.36 (0.78, 2.29)	0.26	1.04 (0.69, 1.51)	0.85	1.11 (0.77, 1.59)	0.56
**IMID[2]**	1.31 (0.78, 2.07)	0.27	0.82 (0.24, 2.08)	0.71	0.64 (0.25, 1.35)	0.29	1.60 (0.91, 2.64)	0.081
**rs2234235, *TAS2R1***	**1.72 (1.13, 2.52)**	**0.007**	0.94 (0.32, 2.20)	0.89	**2.04 (1.25, 3.17)**	**0.003**	1.03 (0.52, 1.81)	0.93
rs2234009, *TAS2R5*	0.92 (0.52, 1.51)	0.75	1.47 (0.52, 3.33)	0.40	1.12 (0.58, 1.98)	0.71	0.94 (0.42, 1.79)	0.87
**rs2234010, *TAS2R5***	**1.71 (1.15, 2.47)**	**0.006**	0.88 (0.26, 2.26)	0.82	1.38 (0.81, 2.23)	0.21	**1.75 (1.01, 2.86)**	**0.033**
rs1726866, *TAS2R38*	0.85 (0.71, 1.01)	0.071	1.00 (0.71, 1.41)	0.99	0.84 (0.68, 1.04)	0.12	0.94 (0.75, 1.18)	0.60
rs34039200, *TAS2R62P*	0.95 (0.77, 1.17)	0.66	1.18 (0.77, 1.76)	0.44	0.91 (0.69, 1.17)	0.45	1.10 (0.83, 1.43)	0.50
rs3851584, *TAS2R14*	0.97 (0.82, 1.15)	0.74	0.92 (0.64, 1.30)	0.62	1.00 (0.81, 1.24)	0.97	0.92 (0.74, 1.16)	0.49
**rs77837442, *TAS2R19***	**0.23 (0.04, 0.71)**	**0.036**	1.43 (0.33, 4.30)	0.57	0.40 (0.07, 1.28)	0.21	0.47 (0.11, 1.28)	0.21
rs117458236, *TAS2R20*	0.85 (0.40, 1.60)	0.65	1.45 (0.42, 3.86)	0.50	0.55 (0.17, 1.33)	0.24	1.54 (0.71, 2.97)	0.23

[1]Logistic regression; OR = Odds Ratio; CI = Confidence Interval.
[2]IMID = immune-mediated inflammatory disease

Table S5 (Continued). Association between *TAS2R* variants and SARS-CoV-2 nucleocapsid antibody in patients with chronic medical conditions and in corresponding control groups, defined as individuals without the specific condition under analysis.

**Characteristic**	**Respiratory disease**				**Immune-mediated inflammatory disease**			
	**Controls (N=5,964)**		**Patients (N=1,511)**		**Controls (N=7,047)**		**Patients (N=428)**	
	**OR (95% CI)[1]**	**p-value**	**OR (95% CI)[1]**	**p-value**	**OR (95% CI)[1]**	**p-value**	**OR (95% CI)[1]**	**p-value**
Age	0.99 (0.98, 1.01)	0.31	0.99 (0.97, 1.02)	0.67	0.99 (0.98, 1.01)	0.36	0.98 (0.91, 1.04)	0.46
Sex (female)	0.87 (0.68, 1.11)	0.25	0.86 (0.53, 1.39)	0.53	0.91 (0.72, 1.13)	0.38	0.49 (0.16, 1.40)	0.19
Smoking	1.07 (0.57, 1.83)	0.82	0.30 (0.05, 1.02)	0.11	0.78 (0.42, 1.33)	0.40	2.91 (0.36, 15.6)	0.25
Dwelling ownership (rent)	0.96 (0.65, 1.38)	0.83	0.82 (0.35, 1.68)	0.61	0.98 (0.69, 1.37)	0.92	0.44 (0.02, 2.70)	0.46
**Number of household residents**	0.98 (0.86, 1.09)	0.75	**1.20 (1.04, 1.40)**	**0.012**	1.05 (0.96, 1.13)	0.19	0.85 (0.39, 1.71)	0.66
Number of vaccine doses	0.96 (0.79, 1.17)	0.70	1.00 (0.69, 1.46)	0.98	0.97 (0.81, 1.15)	0.72	0.96 (0.43, 2.07)	0.92
**Post-secondary education**	0.98 (0.68, 1.46)	0.92	**0.47 (0.25, 0.95)**	**0.026**	0.79 (0.57, 1.11)	0.16	4.40 (0.72, 88.1)	0.19
Diabetes	0.88 (0.63, 1.20)	0.43	1.24 (0.71, 2.10)	0.43	0.99 (0.75, 1.31)	0.96	0.54 (0.14, 1.70)	0.33
Cardiovascular disease	0.96 (0.74, 1.24)	0.74	0.95 (0.57, 1.59)	0.84	0.90 (0.71, 1.14)	0.38	2.45 (0.78, 8.61)	0.14
Respiratory disease	-	-	-	-	1.09 (0.82, 1.42)	0.56	1.04 (0.33, 2.97)	0.94
IMID[2]	1.16 (0.67, 1.88)	0.56	1.20 (0.45, 2.67)	0.68	-	-	-	
**rs2234235, *TAS2R1***	**1.90 (1.26, 2.76)**	**0.001**	0.50 (0.12, 1.38)	0.25	**1.54 (1.03, 2.23)**	**0.026**	1.59 (0.31, 5.96)	0.52
rs2234009, *TAS2R5*	1.08 (0.63, 1.74)	0.76	0.77 (0.19, 2.04)	0.65	1.10 (0.67, 1.69)	0.70	0.00 (0.00, inf)	>0.99
**rs2234010, *TAS2R5***	**1.67 (1.10, 2.44)**	**0.011**	1.16 (0.44, 2.53)	0.73	**1.49 (1.00, 2.14)**	**0.041**	**4.19 (1.06, 15.2)**	**0.031**
rs1726866, *TAS2R38*	0.90 (0.75, 1.07)	0.22	0.81 (0.58, 1.15)	0.24	0.91 (0.77, 1.06)	0.23	0.68 (0.33, 1.39)	0.30
rs34039200, *TAS2R62P*	0.95 (0.77, 1.17)	0.62	1.23 (0.81, 1.81)	0.31	1.00 (0.83, 1.21)	>0.99	0.65 (0.24, 1.60)	0.37
rs3851584, *TAS2R14*	1.00 (0.84, 1.18)	0.96	0.88 (0.61, 1.24)	0.46	0.99 (0.85, 1.16)	0.93	0.55 (0.24, 1.17)	0.14
rs77837442, *TAS2R19*	0.48 (0.15, 1.15)	0.15	0.38 (0.02, 1.77)	0.34	0.51 (0.18, 1.11)	0.14	0.00 (-, -)	>0.99
rs117458236, *TAS2R20*	0.86 (0.40, 1.62)	0.67	1.47 (0.42, 3.92)	0.48	0.91 (0.46, 1.61)	0.76	1.84 (0.22, 10.1)	0.52

[1]Logistic regression; OR = Odds Ratio; CI = Confidence Interval.
[2]IMID = immune-mediated inflammatory disease.

Table S6. Association between *TAS2R* variants and SARS-CoV-2 spike antibody in patients with chronic medical conditions and in corresponding control groups, defined as individuals without the specific condition under analysis.

**Characteristic**	**Diabetes**				**Cardiovascular disease**			
	**Controls (N=5,875)**		**Patients (N=1,600)**		**Controls (N=3,759)**		**Patients (N=3,716)**	
	**OR (95% CI)**[1]	**p-value**	**OR (95% CI)**[1]	**p-value**	**OR (95% CI)**[1]	**p-value**	**OR (95% CI)**[1]	**p-value**
Age	1.00 (0.99, 1.01)	0.89	1.00 (0.98, 1.01)	0.63	1.00 (0.99, 1.01)	0.96	1.00 (0.99, 1.01)	0.82
**Sex (female)**	**1.37 (1.17, 1.60)**	**<0.001**	0.92 (0.69, 1.24)	0.60	**1.38 (1.12, 1.69)**	**0.002**	1.17 (0.97, 1.41)	0.11
Smoking	0.92 (0.63, 1.37)	0.67	1.01 (0.50, 2.10)	0.97	0.83 (0.52, 1.34)	0.42	1.09 (0.68, 1.80)	0.72
Dwelling ownership (rent)	1.01 (0.79, 1.29)	0.94	0.79 (0.53, 1.17)	0.24	1.02 (0.74, 1.42)	0.92	0.89 (0.68, 1.16)	0.39
Number of household residents	1.05 (0.97, 1.14)	0.28	1.02 (0.89, 1.20)	0.74	1.03 (0.94, 1.14)	0.59	1.07 (0.97, 1.20)	0.19
**Number of vaccine doses**	**110 (84.8, 145)**	**<0.001**	**58.9 (39.5, 91.3)**	**<0.001**	**130 (93.9, 185)**	**<0.001**	**64.2 (48.3, 87.4)**	**<0.001**
Post-secondary education	1.09 (0.84, 1.41)	0.49	1.27 (0.85, 1.89)	0.23	1.08 (0.75, 1.54)	0.66	1.20 (0.91, 1.57)	0.19
Diabetes	-	-	-	-	0.80 (0.59, 1.10)	0.16	0.93 (0.76, 1.13)	0.45
Cardiovascular disease	0.86 (0.73, 1.02)	0.080	1.02 (0.73, 1.42)	0.92	-	-	-	-
Respiratory disease	0.99 (0.81, 1.21)	0.90	0.76 (0.54, 1.07)	0.12	1.02 (0.78, 1.34)	0.87	0.88 (0.71, 1.10)	0.25
**IMID**[2]	**0.63 (0.45, 0.88)**	**0.006**	0.68 (0.40, 1.17)	0.16	**0.59 (0.38, 0.93)**	**0.021**	**0.70 (0.49, 1.01)**	**0.055**
rs2234235, *TAS2R1*	0.80 (0.58, 1.11)	0.17	0.59 (0.34, 1.04)	0.067	0.79 (0.54, 1.19)	0.25	0.70 (0.48, 1.03)	0.066
**rs2234009, *TAS2R5***	1.13 (0.81, 1.60)	0.47	**2.19 (1.17, 4.24)**	**0.017**	1.26 (0.81, 2.02)	0.32	1.36 (0.92, 2.05)	0.13
**rs2234010, *TAS2R5***	1.07 (0.78, 1.48)	0.68	**2.15 (1.13, 4.16)**	**0.022**	1.24 (0.82, 1.91)	0.32	1.28 (0.87, 1.91)	0.22
rs1726866, *TAS2R38*	1.02 (0.91, 1.14)	0.78	1.13 (0.92, 1.39)	0.25	1.03 (0.89, 1.20)	0.69	1.04 (0.91, 1.18)	0.60
**rs34039200, *TAS2R62P***	0.91 (0.80, 1.04)	0.16	**0.72 (0.56, 0.92)**	**0.008**	**0.84 (0.71, 1.00)**	**0.051**	0.87 (0.75, 1.02)	0.095
rs3851584, *TAS2R14*	1.02 (0.91, 1.14)	0.72	1.05 (0.86, 1.29)	0.62	1.03 (0.89, 1.20)	0.66	1.01 (0.89, 1.16)	0.84
**rs77837442, *TAS2R19***	1.10 (0.72, 1.72)	0.68	**0.44 (0.19, 1.02)**	**0.054**	1.31 (0.71, 2.53)	0.41	0.75 (0.47, 1.22)	0.25
rs117458236, *TAS2R20*	0.95 (0.65, 1.42)	0.81	0.92 (0.46, 1.89)	0.81	0.80 (0.49, 1.33)	0.38	1.05 (0.66, 1.71)	0.83

[1]Logistic regression; OR = Odds Ratio; CI = Confidence Interval.
[2]IMID = immune-mediated inflammatory disease.

Table S6 (Continued). Association between *TAS2R* variants and SARS-CoV-2 spike antibody in patients with chronic medical conditions and in corresponding control groups, defined as individuals without the specific condition under analysis.

**Characteristic**	**Respiratory disease**				**Immune-mediated inflammatory disease**			
	**Controls (N=5,964)**		**Patients (N=1,511)**		**Controls (N=7,047)**		**Patients (N=428)**	
	**OR (95% CI)[1]**	**p-value**	**OR (95% CI)[1]**	**p-value**	**OR (95% CI)[1]**	**p-value**	**OR (95% CI)[1]**	**p-value**
Age	1.00 (0.99, 1.01)	0.99	1.00 (0.98, 1.01)	0.62	1.00 (0.99, 1.01)	>0.99	0.98 (0.95, 1.02)	0.28
**Sex (female)**	**1.33 (1.14, 1.56)**	**<0.001**	1.01 (0.74, 1.38)	0.94	**1.25 (1.08, 1.44)**	**0.003**	1.24 (0.69, 2.21)	0.47
Smoking	1.12 (0.75, 1.71)	0.58	0.60 (0.32, 1.13)	0.11	0.87 (0.62, 1.24)	0.43	2.21 (0.56, 9.47)	0.27
Dwelling ownership (rent)	0.86 (0.69, 1.09)	0.22	1.19 (0.76, 1.88)	0.45	0.94 (0.76, 1.16)	0.54	0.86 (0.36, 2.04)	0.72
**Household size**	1.02 (0.95, 1.10)	0.64	**1.18 (1.00, 1.41)**	**0.053**	1.05 (0.98, 1.13)	0.21	1.10 (0.76, 1.61)	0.62
**Number of vaccine doses**	**98.0 (76.5, 128)**	**<0.001**	**75.5 (48.4, 124)**	**<0.001**	**95.5 (76.2, 122)**	**<0.001**	**54.3 (25.4, 135)**	**<0.001**
**Post-secondary education**	**1.26 (0.99, 1.59)**	**0.058**	0.88 (0.52, 1.47)	0.63	1.11 (0.89, 1.39)	0.36	**2.50 (1.00, 6.43)**	**0.052**
**Diabetes**	0.96 (0.79, 1.17)	0.70	**0.74 (0.52, 1.05)**	**0.091**	0.89 (0.75, 1.06)	0.20	1.12 (0.58, 2.16)	0.74
**Cardiovascular disease**	0.92 (0.78, 1.09)	0.33	0.82 (0.59, 1.13)	0.22	0.89 (0.76, 1.03)	0.12	1.12 (0.60, 2.07)	0.73
**Respiratory disease**	-	-	-	-	0.93 (0.78, 1.10)	0.39	0.93 (0.48, 1.77)	0.81
**IMID[2]**	**0.62 (0.45, 0.86)**	**0.004**	0.77 (0.44, 1.36)	0.36	-	-	-	-
**rs2234235, *TAS2R1***	0.84 (0.62, 1.16)	0.29	**0.48 (0.27, 0.85)**	**0.012**	0.77 (0.58, 1.03)	0.078	**0.37 (0.13, 1.04)**	**0.058**
**rs2234009, *TAS2R5***	1.32 (0.95, 1.85)	0.10	1.50 (0.75, 3.10)	0.26	**1.38 (1.01, 1.91)**	**0.044**	1.04 (0.34, 3.25)	0.95
rs2234010, *TAS2R5*	1.29 (0.94, 1.79)	0.12	1.14 (0.61, 2.21)	0.68	1.22 (0.91, 1.66)	0.18	1.40 (0.47, 4.32)	0.55
rs1726866, *TAS2R38*	1.03 (0.92, 1.15)	0.64	1.03 (0.83, 1.28)	0.78	1.04 (0.94, 1.15)	0.50	1.00 (0.65, 1.54)	0.99
**rs34039200, *TAS2R62P***	**0.86 (0.76, 0.99)**	**0.030**	0.84 (0.65, 1.10)	0.20	**0.87 (0.77, 0.98)**	**0.027**	0.70 (0.41, 1.16)	0.17
rs3851584, *TAS2R14*	1.02 (0.91, 1.14)	0.74	1.06 (0.86, 1.32)	0.58	1.04 (0.94, 1.15)	0.50	0.86 (0.57, 1.30)	0.48
rs77837442, *TAS2R19*	0.91 (0.59, 1.41)	0.66	1.13 (0.54, 2.47)	0.76	0.90 (0.61, 1.34)	0.59	1.02 (0.29, 3.78)	0.98
rs117458236, *TAS2R20*	0.90 (0.62, 1.34)	0.60	1.05 (0.52, 2.20)	0.90	1.01 (0.71, 1.46)	0.96	0.76 (0.23, 2.41)	0.64

[1]Logistic regression; OR = Odds Ratio; CI = Confidence Interval.
[2]IMID = immune-mediated inflammatory disease.

SNP	Wild Type mRNA Secondary Structure	Mutant mRNA Secondary Structure	Predicted Base Pair Probabilities
Rs2234010 *TAS2R5* A>G	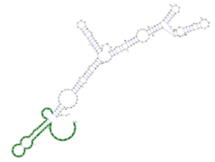	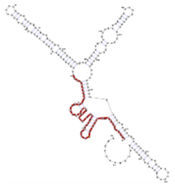	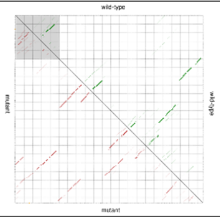
rs2234235 *TAS2R1* U>C	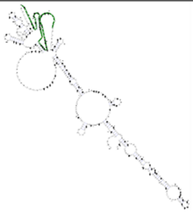	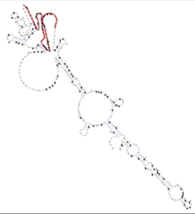	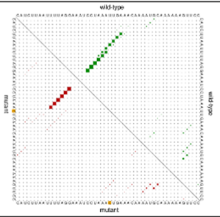
rs117458236 *TAS2R20* G>A	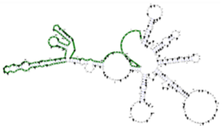	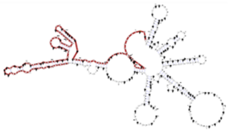	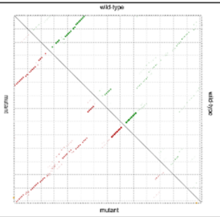

Figure S3. Predicted mRNA secondary structures and base pair probabilities calculated by RNAsnp. In each matrix, the upper triangle represents base pair probabilities for the wild-type sequence, and the lower triangle represents probabilities for the mutant sequence. Red and green dots indicate base pairs whose probabilities differ between the two alleles. The results reveal structural changes in mRNA for the variants, with the rs2234010 in *TAS2R5* predicted to have the most notable impact.
